# Image-based facial emotion recognition using convolutional neural network on emognition dataset

**DOI:** 10.1038/s41598-024-65276-x

**Published:** 2024-06-23

**Authors:** Erlangga Satrio Agung, Achmad Pratama Rifai, Titis Wijayanto

**Affiliations:** Department of Mechanical and Industrial Engineering, Universitas Gadjah Mada, Yogyakarta, Indonesia

**Keywords:** Facial emotion recognition, Convolutional neural network, Deep learning, Emognition dataset, Human behaviour, Computer science, Information technology

## Abstract

Detecting emotions from facial images is difficult because facial expressions can vary significantly. Previous research on using deep learning models to classify emotions from facial images has been carried out on various datasets that contain a limited range of expressions. This study expands the use of deep learning for facial emotion recognition (FER) based on Emognition dataset that includes ten target emotions: amusement, awe, enthusiasm, liking, surprise, anger, disgust, fear, sadness, and neutral. A series of data preprocessing was carried out to convert video data into images and augment the data. This study proposes Convolutional Neural Network (CNN) models built through two approaches, which are transfer learning (fine-tuned) with pre-trained models of Inception-V3 and MobileNet-V2 and building from scratch using the Taguchi method to find robust combination of hyperparameters setting. The proposed model demonstrated favorable performance over a series of experimental processes with an accuracy and an average F1-score of 96% and 0.95, respectively, on the test data.

## Introduction

Humans use emotions to express their feelings to others and as a communication tool to convey information. Emotions reflect human mood in the form of a psychophysiological condition of a human. Emotions result from human interactions and internal or external factors^1^. Dynamic changes in emotion play an important role in human life because they directly affect most of the daily activities and habits carried out by humans^2^. Emotions are the dominant driver of decisions made by humans^3^. Positive emotions result in the formation of good communication and increase human productivity. Meanwhile, negative emotions can harm both mental and physical conditions. Therefore, an automated system based on human emotions is important for continuing to develop^4^.

Humans can express emotions through hands, voice, gestures, and facial expressions, with 55% of emotions conveyed through facial expressions^5^. The human face displays information cues that are relevant to provide expression of an emotional state or behavior. Facial expressions play an important role in human communication, as they help us understand the intentions of others. Hence, facial recognition emerges as an important domain in understanding human emotion. Among various facial recognition techniques, facial emotion recognition (FER) has seen substantial advancement^6^. Using machine learning, FER can help humans distinguish emotions through facial expressions by analyzing images or video data to obtain information about emotional states^7^, which is important for social interaction because it can help humans understand the feelings and intentions of others. FER is commonly used in various fields such as health, education, labor, robotics, communication, psychology, and others^8^.

Recent advancements in FER-based automation systems can be classified into two main parts of feature generation: conventional extraction and automatic extraction via deep neural networks^9^. While the conventional approach holds an advantage in computation time and is commonly used for real-time classification problems^10^, this approach lacks flexibility as it requires predefined feature extraction and classifiers^11^. As such, deeper knowledge of the feature extraction and classifier is required to fetch meaningful and good features for models’ input without discarding important information. This issue can be an obstacle to developing and implementing the detection models. On the other hand, an automated approach employing deep learning algorithms, such as the convolutional neural network (CNN), reduces or eliminates dependencies from other models and/or existing preprocessing techniques by carrying out end-to-end learning directly from input data^12^. However, CNNs require extensive data to obtain a higher level of classification^13^. Researchers worldwide have done much research by building the CNN model to solve the FER problem. Researchers have used various types of image data-based datasets as input data for the constructed models, such as Facial Emotion Recognition 2013 (FER 2013) and Extended Cohn-Kanade Dataset (CK +). However, the entire dataset still focuses on the 7 basic human emotions, so further development is needed to solve the FER problem with a more varied number of emotion classes.

This study is motivated by the need to address the limitations of the existing FER systems that predominantly recognize a limited set of basic emotions. Utilizing the Emognition dataset, which encompasses ten distinct emotions: neutral, amusement, anger, awe, disgust, enthusiasm, fear, liking, sadness, and surprise^14^, this study aims to develop FER models capable of handling a wider spectrum of human emotions. The Emognition dataset not only includes common emotions but also introduces four new emotion classes: awe, enthusiasm, amusement, and liking, providing a richer foundation for enhancing FER applications in areas such as mobile application development, education, product marketing, and tourism management. For example, the inclusion of the amusement emotion improves interaction with entertainment devices like games and movies^15^. Recognizing enthusiasm can help educators enhance learning environments and manage student engagement effectively^16^. In other fields, the emotion of enthusiasm can also be used to determine the suitability of the workload given to a worker, the awe emotion can significantly increase consumer willingness to share^17^, and liking emotion also has a role in shaping consumer preferences and brand affinity in marketing. Despite its potential, using the Emognition dataset in FER models is relatively unexplored, representing a significant gap in current research that this study seeks to address.

This study also explores whether CNNs, trained on the Emognition dataset^14^, can more effectively classify a more diverse range of emotions. This involves assessing the benefits of processing image data extracted from video sequences compared to direct video inputs, which could potentially allow for selecting relevant data to use and discarding irrelevant data, thus optimizing the performance of the FER models. Overall, this study aims to improve the accuracy and applicability of FER systems and broaden the scope of emotions that these systems can recognize. Such advancements in emotion recognition technology could have significant implications across various aspects of human life, including social interactions, mental health, education, and employment. This ongoing development of FER technology highlights its critical role as a necessary knowledge domain in the modern world.

The remainder of this paper is as follows. In the next section, previous related work is explained. In Sect. “Methodology”, the proposed method and the background theories are described. In Sect. “Experimental setup”, experimental works and obtained results are examined and analyzed. In the last section, conclusions and future works are discussed.

## Related work

Research in FER has evolved through conventional and automated approaches involving various datasets and methodologies. Thus far, numerous studies have utilized datasets such as Cohn-Kanade (CK)^18^, Extended Cohn-Kanade (CK +)^19^, Facial Expression Recognition 2013 (FER 2013)^20^, Japanese Female Facial Expression (JAFFE)^21^, FACES^22^, and Radboud Faces Database (RaFD)^23^. These datasets primarily include six to eight basic emotion classes. For example, CK and CK + have seven emotional classes: neutral, anger, contempt, disgust, fear, happiness, and sadness^24^. Similarly, FER 2013 and JAFFE introduce seven classes with slightly different categories: neutral, angry, disgusted, fearful, happy, sad, and surprised^24^. While the datasets as mentioned earlier introduced seven classifications, FACES introduced six categories of emotions: neutral, sad, disgust, fear, anger, and happy^22^, while RaFD has eight emotional classes: anger, disgust, fear, happiness, sadness, surprise, disdain, and neutral. Albeit these datasets have provided insightful data, the emotions covered are considerably basic emotions and less complex.

Recently, Saganowski et al.^14^ introduced the Emognition dataset, which includes ten distinct emotions: neutral, amusement, anger, awe, disgust, enthusiasm, fear, liking, sadness, and surprise. In addition to these emotions, the dataset offers several enhancements: it captures physiological signals, represents emotional states using both discrete and dimensional scales, and highlights differences among positive emotions. These improvements facilitate emotion recognition from both facial expression analysis and physiological perspectives, accommodating variances that might occur with specific emotions. While several studies have utilized the physiological signals from the Emognition dataset to classify emotions (e.g.,^25–28^), there has been relatively little research examining the facial expression data within the same dataset for emotion recognition and classification (e.g.,^29^. This gap highlights a key area for further investigation, aiming to fully leverage the dataset's capabilities in enhancing FER technologies.

Concerning FER techniques, researchers use several conventional methods to extract features from input image data. Some of these methods have been applied in several publications, including cropping faces and converting them into grayscale images^30^, using an optical flow-based approach^31^, and using a histogram of oriented gradients (HOG)^32^. In addition, several publications use an automated approach in extracting features based on the CNN algorithm, including the development of a model with an architectural configuration from scratch^33^, transfer learning with MobileNet^34^**,** MobileNet-V2^35^**,** VGG19^36^, DenseNet121^37^, and others.

In the conventional approach, Gupta^30^ preprocessed the CK and Extended CK + datasets by cutting the facial region using the HAAR filter from OpenCV and then converting them into grayscale images. In addition, the detection of landmark points on the face is normalized at each point. The random sample technique in the training data distribution is applied with a ratio of 80% training data and 20% validation data. The training process is carried out with the support vector machine classifier model and obtains an accuracy of 93%. Using the same type of classifier, Anthwal and Ganotra^31^ performed dense optical flow calculations on facial images to extract vertical and horizontal components. Preprocessing in this study was carried out using the Viola-Jones algorithm to cut the facial area and resize it to a grayscale image with a resolution of 256 × 256. Using Extended Cohn-Kanade (CK +) as a dataset, the best results were obtained for 6 class categories (excluding the contempt class) with 90.52% accuracy. In contrast, for 7 classes, only 83.38% accuracy was achieved, indicating that the classifier model performed better when trained on 6 class categories compared to 7 class categories.

Using the JAFFE and Cohn-Kanade (CK) datasets, Supta et al.^32^ built an FER system based on the HOG and support vector machine (SVM). Preprocessing is carried out on the detected parts using histogram equalization techniques and image sharpening to reduce lighting effects. Then, HOG extracts distinctive image features from faces and combines them into feature vectors. Finally, SVM is used to classify expressions using polynomial kernel functions. The proposed system is evaluated on JAFFE and CK data and shows that the proposed FER system provides up to 97.62% and 98.61% accuracy for JAFFE and CK data, respectively.

The conventional feature extraction method for FER requires complex image preprocessing and manual feature extraction, which take a long time^38^. Manually extracted features depend heavily on the previous knowledge of the researchers. This causes the resulting features to be exposed to high bias and causes the loss of implicit patterns. The effectiveness of the extracted features also depends on how well the manual feature extraction technique is used. In contrast, an automated approach based on deep learning is very good at classifying images but requires extensive data to train and perform recognition efficiently^13^.

Extensive data requirements are one of the crucial factors in the context of deep learning model development. Ramalingam and Garzia^33^ developed the CNN algorithm using a combination layer of convolution, rectified linear unit (ReLU), pooling twice for feature extraction, and ending with a fully connected layer. This CNN algorithm achieved an accuracy of only 60% on the FER 2013 dataset, which included 35,887 sample images. One deep learning approach that can improve training accuracy is using transfer learning techniques. This technique takes advantage of features learned by models trained on ImageNet to overcome the problem of the lack of large datasets available online^35^.

Ramalingam and Garzia^33^ also used transfer learning with the pretrained VGG16 model. In the FER2013 dataset, the accuracy of this transfer learning algorithm reaches 78%, so there is an increase in accuracy of 18% compared to the CNN model without transfer learning. With the same dataset, Raja Sekaran et al.^13^ implemented transfer learning using AlexNet as a pretrained model. This research also implements an early stopping mechanism to overcome the problem of overfitting AlexNet. The proposed model only requires preprocessing in the form of image conversion to grayscale to reduce the effects of lighting and human skin color on the classification results. The model managed to achieve 70.52% accuracy for the FER dataset.

In FER 2013, Abdulsattar and Hussain^37^ developed six well-known deep learning models for FER problems. These models are MobileNet, VGG16, VGG19, DenseNet121, Inception-v3, and Xception. Model performance was evaluated and compared using transfer learning and fine-tuning strategies on the FER2013 dataset. In transfer learning, all layers in the pretrained model are frozen or not retrained. However, in fine-tuning, all layers in the pretrained model are retrained. The results show that the fine-tuning strategy performs better than transfer learning, with differences ranging from 1 to 10%. The VGG16 pretrained model achieved the highest accuracy with a maximum accuracy of 0.6446.

Transfer learning using VGG16 was also carried out by Oztel et al.^39^ using the RaFD dataset. The treatment of the VGG16 pretrained model was divided into two scenarios: with transfer learning and without transfer learning. The model without transfer learning was modified on the 39th and 41st layers of the model structure and a random weight was placed on the model layer, while the model with transfer learning kept the model structure intact. The VGG16 scenario with transfer learning produced the best accuracy compared to the VGG16 scenario without transfer learning. The less-optimal results in the VGG16 scenario without transfer learning are due to a lack of training data and input data imbalance.

Gondkar et al.^35^ also carried out research using transfer learning on CK + . The models used in the transfer learning technique are Xception, VGG16, VGG19, ResNet50, ResNet101, ResNet152, ResNet50V2, ResNet101V2, ResNet152V2, InceptionV3, InceptionResNetV2, MobileNet, MobileNetV2, and DenseNet121. A comparative analysis of these models was performed using various evaluation metrics, such as model size, training accuracy, validation accuracy, training loss, and validation loss. The results showed that the pretrained models ResNet50V2, ResNet101V2, ResNet152V2, and MobileNet achieved training and validation accuracy of more than 90%. Most pretrained models have demonstrated outstanding performance, with ResNet101V2 achieving a training accuracy of 93.08% and a validation accuracy of 92.87%. MobileNet achieved training and validation accuracies higher than 90%. Regarding model size, MobileNet was the smallest yet more efficient than most other models.

Undeniably, the type of preprocessing used in the input data can impact the quality of the resulting model. This is evidenced by the preprocessing experiment conducted by Sajjanhar et al.^36^. In that study, three treatments were applied in the CK + , JAFFE, and FACES datasets. The first preprocessing step involved maintaining the region of interest (ROI) or by cutting the face area from the image. The second preprocessing step was performed by calculating the difference between the gray level intensities of the ROI image pixels at neutral and peak expressions. The third preprocessing step was performed by forming a local binary pattern (LBP) from the image. These three types of preprocessing were tested on the CNN algorithm and resulted that the second preprocessing succeeded in providing the best accuracy compared to other types of preprocessing with 85.19% in CK + data, 65.17% in JAFFE data, and 84.38% in FACES data.

Agobah et al.^34^ applied transfer learning using MobileNetV1 across multiple datasets for training, validation, and testing. They optimize CNN training by combining center loss and softmax loss, using the FER 2013 dataset for training and the JAFFE and CK + datasets for validation and testing. This addition improved accuracies on CK + and JAFFE by 2.94% and 5.08%, respectively, with JAFFE achieving 96.43% precision and 95.24% recall and F1 score. While increasing the number of classes in the CK + dataset initially reduced accuracy due to complexity and data limitations, using a larger dataset significantly enhanced performance, raising accuracy by 4.41% over a smaller dataset. However, Agobah et al.^34^ highlighted that some misclassifications occurred due to the inherent complexity of distinguishing emotions like anger and sadness.

Meena et al.^40^ explored the use of CNN for sentiment identification on facial expression in the CK + and FER-2013 datasets. Several architectures were developed to evaluate the efficiency of the proposed models in those datasets. The models were categorized into two types based on the data classes: CNN-3 model considered positive, negative, and neutral expressions, while CNN-7 model considered happy, neutral, sad, surprise, fear, angry, and disgust expressions. The CNN-3 model yielded accuracy at 79% and 95% for the FER-2013 and CK + databases, respectively. Meanwhile, the CNN-7 model resulted on slightly lower accuracy at 69% and 92% for the same datasets.

Recent FER studies explored advanced models for emotion recognition from video data^29,41^. Bilotti et al.^41^ developed a multimodal CNN approach integrating facial frames, optical flow, and Mel Spectrograms, which achieved impressive accuracies of approximately 95% on the BAUM-1 and 95.5% on the RAVDESS datasets. In contrast, Manalu and Rifai^29^ focused on hybrid CNN-RNN models, comparing a custom model with transfer learning models based on InceptionV3 and MobileNetV2 architectures. Their custom model achieved a maximum accuracy of 63%, less than the 66% by InceptionV3-RNN but higher than the 59% by MobileNetV2-RNN, with the custom model also offering significantly quicker processing times. Both studies highlight the potential of combining different data inputs and model architectures to enhance the accuracy and efficiency of facial expression recognition systems.

Overall, the studies above show the effectiveness of using the transfer learning method in building models by considering the limited data availability in the FER domain. Several pre-trained models are also used to obtain optimal accuracy on the FER, JAFFE, CK + , CK, FACES, and RaFD datasets. However, from the various studies conducted, no research has discussed the application of CNN-based deep learning using Emognition datasets. Considering that the Emognition dataset covers a more significant number of emotions, some of which have never been explored before, this study develops FER models using CNN to address the Emognition dataset. Besides exploring the transfer learning and fine-tuning strategy, this study also proposes a novel network with aims to develop a more efficient model for FER. Building a CNN from scratch for facial emotion recognition provides a deep understanding of the network’s inner workings, allowing for customization and optimization of the architecture to the specifics of the task. Since the FER images have not been covered by standard pre-trained models, it is worth developing a full learning model which can be customized according to the problem specification.

## Methodology

In this work, we use different types of methods for building the CNN model: transfer learning using MobileNet-V2 and Inception-V3 with fine-tuning strategies and building models from scratch by designing a new network with better efficiency. We also apply a serial type of preprocessing in the Emognition dataset to adjust with the research goal.

### Data pre-processing

**Figure 1:**
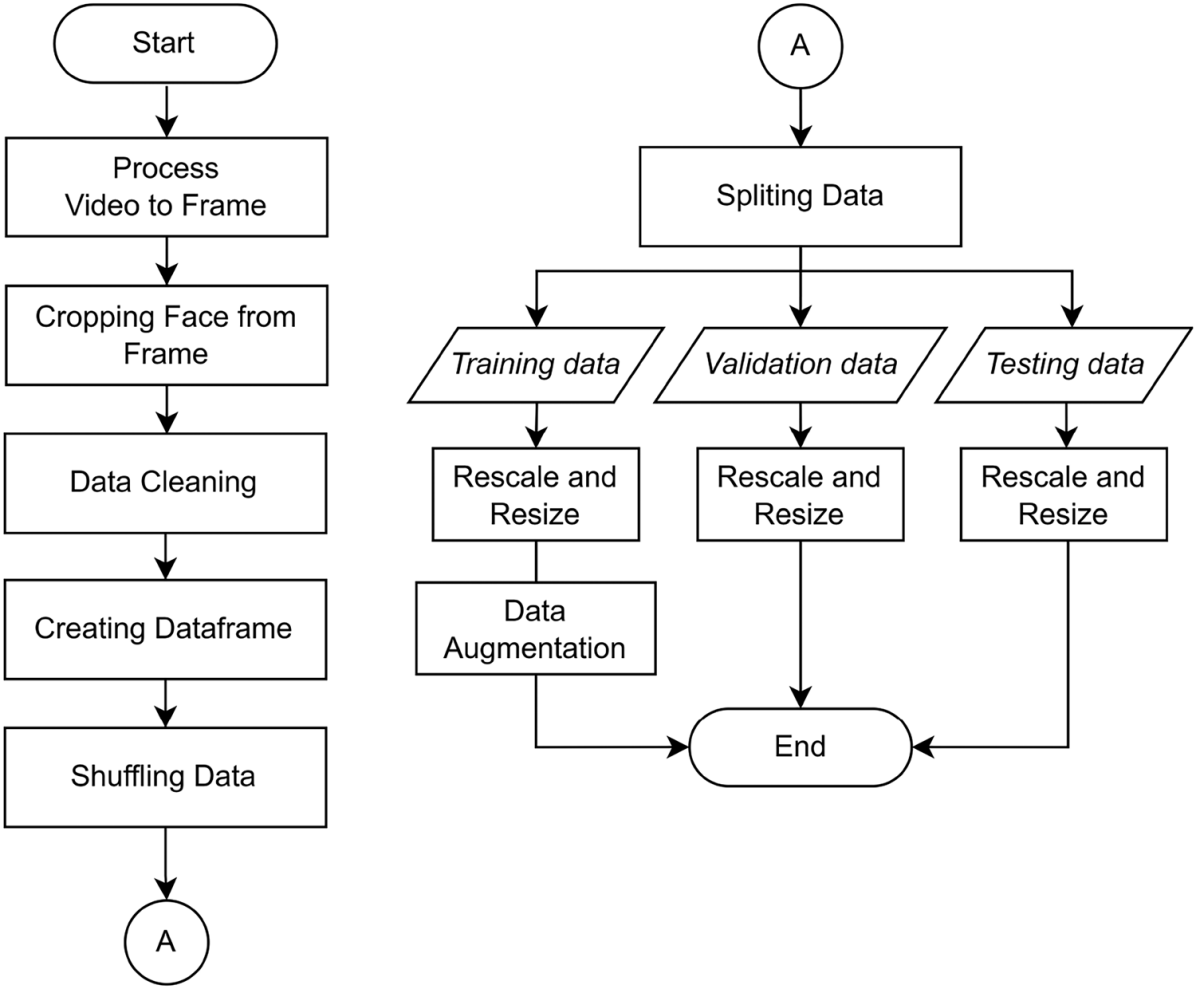
Stages of data pre-processing.

This process is carried out in several sequential stages: process video to frame, face cropping from frame, data cleaning, data splitting, rescaling, resizing, and data augmentation, as depicted in Fig. 1. Following the selected deep learning method, the half-body video data is transformed into image data (frame sequences). This task is performed in the Process Video to Frame stage. Once image data in video frames is obtained, the facial region is automatically detected and then cropped within those frames. This activity took place in the Face Cropping from Frame stage.

Upon obtaining the facial data, data cleaning is performed by retaining facial images corresponding to emotions while discarding those not accurately representing the intended emotions for their respective classes. During the data input process into the model, a dataframe is employed to enhance data management with greater flexibility and transparency. By utilizing the created data frame, shuffling is performed to the data to achieve a more balanced data distribution. The data are then split into training, validation, and test datasets after the shuffling. The training and validation data play a direct role in training the model, while the test data was solely involved in the testing process.

Subsequently, we apply a rescaling process to transform pixel values of the input images into a range between 0 and 1, and resizing was performed to standardize the input image dimensions. These rescaling and resizing procedures are undertaken to conform to the input requirements of the CNN model. The augmentation techniques are also employed to diversify the dataset, reducing the overfitting risk.

### Transfer learning approach

In the transfer learning of proposed models, the pre-trained weight of the feature extraction layer from both MobileNet-V2 and Inception-V3 are called and then fine-tuning on several layers of the pre-trained model are performed. The choice of Inception-V3 is based on its high accuracy in previous studies. Inception-V3 introduces more complex and efficient architectural designs, including asymmetric convolutions. This means it uses convolutions of different sizes within the same module, allowing it to capture patterns over various spatial hierarchies more effectively. As a result, the network can learn more complex features with fewer parameters, reducing the risk of overfitting. However, Inception-V3 still has more than 24 Million parameters which may require longer training time. Meanwhile, MobileNet-V2 is selected for its small size with around 3.4 million parameters and relative good accuracy, making it suitable for application in smaller devices. It uses depthwise separable convolutions as its basic building block, significantly reducing the number of parameters and computational cost compared to traditional convolutional layers without a substantial loss in accuracy. MobileNet-V2 also uses architectural feature known as the inverted residual structure with linear bottlenecks. This design optimizes the flow of information through the network, ensuring that the model remains lightweight while still capturing essential features necessary for accurate predictions albeit not as powerfull as Inception-V3 which can capture more complex features.

Fine-tuning is achieved by deactivating all pre-trained weights and conducting training with several epochs, then we activate half of the pre-trained model and continue further training with the same number of epochs. As such, the training process of the transfer learning model is divided into two scenarios: scenario 1 and scenario 2, in which scenario 1 includes the stage before fine-tuning, and scenario 2 contains the fine-tuning process. Dense layers are also added with a certain number of unit neurons. These transfer learning and fine-tuning stages are adopted based on research from Elgendy^42^. The training process of the model using the transfer learning method conducted in two stages is described as follows:

#### Scenario 1

In this scenario, all convolutional layers of the selected pretrained model are frozen (freezing), and the classification head from the pretrained model is not utilized. A new classification head is then added, tailored to the case of emotion expression classification, and training is conducted. By freezing the pretrained model, the weights in the convolutional network are not updated during the training process. This scenario aims to train only the classification head of the model while maintaining the weights that have been trained in the pretrained model.

#### Scenario 2

Unlike the first scenario, in this scenario, the pretrained model is partially unfrozen at the last 50% of its layers. Training then continues from where it left off in Scenario 1. Here, the first scenario is executed in the first half of the training process, in which if there are 100 epochs, then the first 50 epochs are dedicated for the first stage. Afterward, the training process is continued using the second scenario for the rest of epochs. The main focus here is to train a portion of the layers in the convolutional network to better suit the case of emotion expression classification. The choice to unfreeze the last 50% of layers is because these layers contain features that are specific to the data trained on the previous dataset.

The use of two stage training process using scenarios 1 and 2 of the transfer learning model is to enhance the effectiveness of the transfer learning process. In scenario 1, the focus is on leveraging the pretrained model, which contains previously trained weights. However, as additional layers are added, these weights become more specific to the features of the previous dataset. Therefore, in scenario 2, there is an effort to retrain part of the pretrained model on the last 50% of its layers to align with the current case. This effort optimizes the weights of the pretrained model for better utilization in the transfer learning process.

### MobileNet-V2

MobileNet is a very lightweight image classification model with minimal operations initiated by Sandler et al.^43^. MobileNet has three variants there are MobileNet-V1, MobileNet-V2, and MobileNet-V3. In this study, we use MobileNet-V2 as one of the pre-trained models in the transfer learning process. MobileNet-V2 is the smallest model in size and has the second fastest GPU processing time after MobileNet-V1 on ImageNet dataset. In addition, this model has greater both top-1 accuracy and top-5 accuracy than MobileNet-V1. This model is also composed of parameters with the smallest number compared to other existing models.

**Figure 2:**
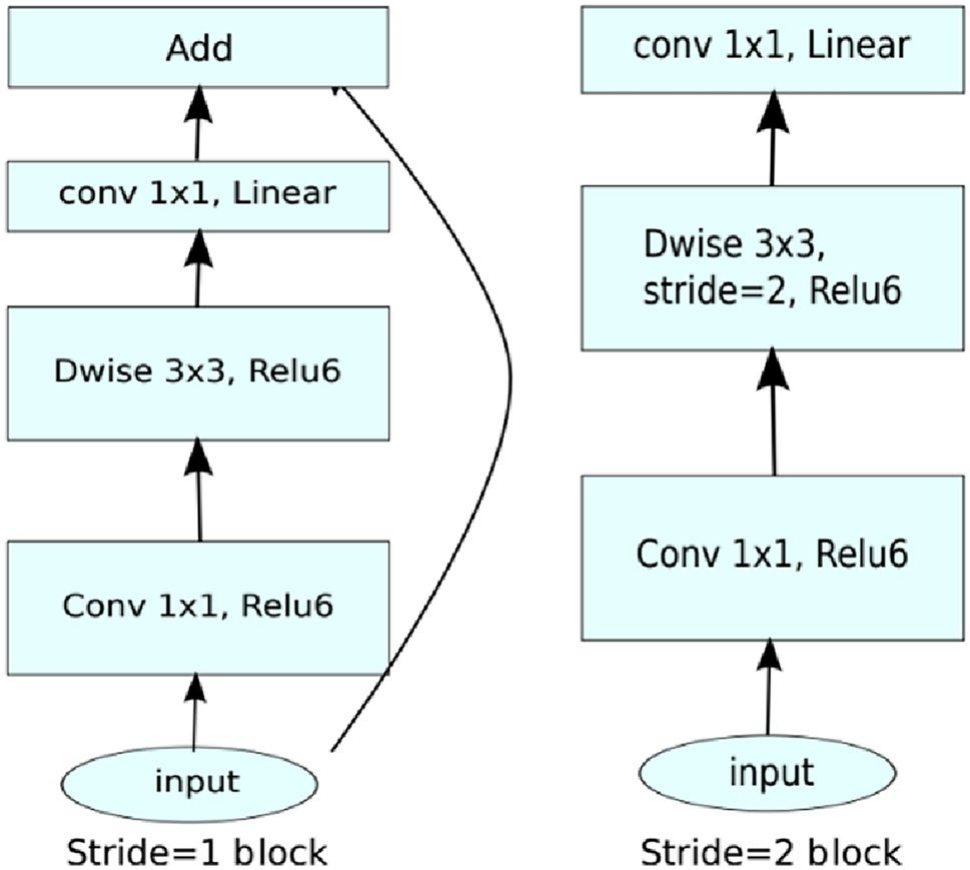
MobileNet-V2 architecture^44^.

MobileNet-V2 has two residual blocks with a stride of 1 and a second block with a stride of 2. Each block consists of 3 layers of 1 × 1 pointwise layer, depthwise layer, and 1 × 1 linear convolution layer with ReLU6 activated. The MobileNet-V2 architecture is demonstrated in Fig. 2.

#### Inception-V3

Inception-V3 is the successor of Inception-V2 and Inception-V1^35^, initiated by Szegedy et al.^45^. This model consists of five 5 × 5 convolution inception modules replaced by two 3 × 3 convolution layers and an efficient grid reduction block to reduce the number of parameters without sacrificing the overall model efficiency. In addition, four 7 × 7 convolution inception modules are replaced with two 1 × 7 and 7 × 1 convolution layers, followed by another grid reduction block.

**Figure 3:**
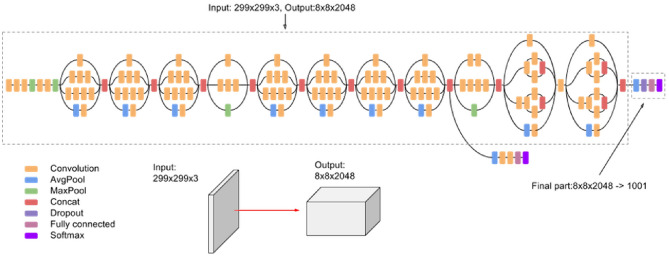
Inception-V3 architecture^46^.

Inception-V3 has an additional classifier connected to the ends of these 4 inception modules. At the end of the model, two inception modules used 3 × 1 and 1 × 3 convolution layers in parallel to increase dimensionality. Then it is connected to the average pooling layer, fully connected layer, dropout, and softmax to produce output. Compared to MobileNet-V2, Inception-V3 has a greater number of parameters with better top-1 accuracy and top-5 accuracy than MobileNet-V2 on ImageNet. The Inception-V3 architecture is shown in Fig. 3.

#### Full learning approach

In the full learning approach, where the model is constructed from the ground up, we employ multiple feature extraction layers. Each of these layers is composed of a convolutional layer paired with a subsequent pooling layer. The convolutional layers vary in the quantity of filters employed and each has a predefined filter size. For the pooling layers, we utilize max pooling. Additionally, a flatten layer is incorporated to transform the feature matrix into a vector form. To diminish the risk of overfitting during the training phase, a dropout layer is also integrated into the model.

**Figure 4:**
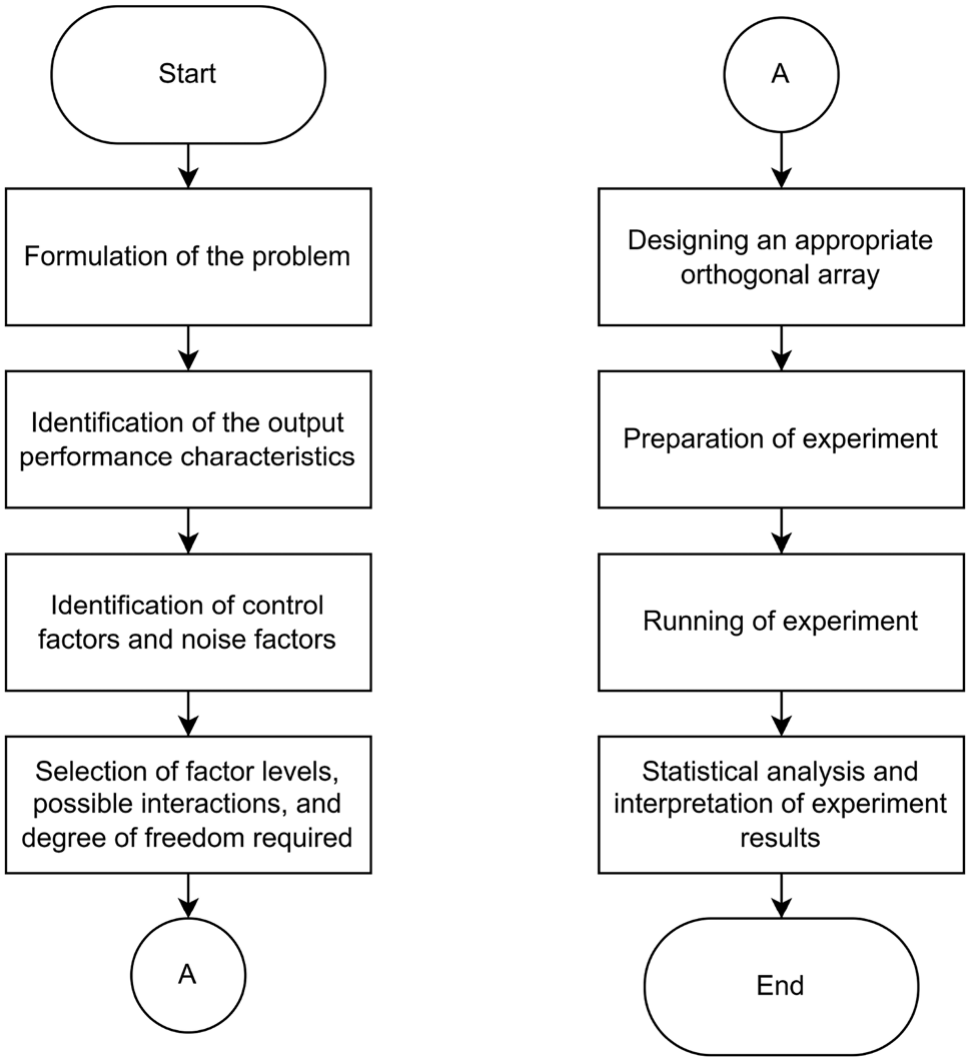
Steps in Design of Experiment using Taguchi Method.

To optimize the result, a design of experiment (DoE) using the Taguchi method is performed to find a robust combination of the number of feature extraction layers and the number of epochs to be used in the build model of full learning technique. The stages of the design of the experiment using the Taguchi method are shown in Fig. 4.

The process begins with the clear definition of the problem at hand, which in this case is to determine a robust combination of the number of feature extraction layers and epochs for the model. Following this, the output performance characteristics that will gauge the success of the model. The output characteristic to be compared is the validation accuracy at the end of each epoch since it enables direct comparisons between different models. The higher the accuracy level on the validation data, the better the model performs.

The next step involves pinpointing the control factors, which include the network architecture details (number of convolutional layers) and training parameter (number of epoch). The number of convolutional layers determines the depth of feature extraction, which can affect a model's ability to learn from complex data. More layers can capture intricate patterns but also risk overfitting. The number of epochs affects how well the model learns from the data; too few epochs can lead to underfitting, while too many may lead to overfitting. Both factors directly influence the model’s learning capacity and generalization to new data, making them critical control factors for optimizing performance.

Moving on, the levels of each control factor to test are selected, considers potential interactions between them, and ensures the appropriate degrees of freedom for the experiment’s statistical validity. Here, each factor has 3 levels (number of convolutional layers: 4, 5, 6, and number of epochs: 50, 100, 150). The most efficient combination was achieved through this by conducting 9 runs in total, hence utilizing an L9 orthogonal array. Afterward, the experiments are executed using the same platform and device to minimize noise. Statistical analysis is conducted by analyzing the value of the S/N ratio from the experimental results obtained. Since the goal of this experiment is to find a combination of factors that can maximize highest validation accuracy value of the expected output, the type of S/N ratio chosen is the S/N ratio larger is better. The output of the DoE is the best combination of number of convolutional layers and number of epoch which are then used for building and training full learning model.

### Evaluation criteria

After constructing the model using both techniques, this study analyze the outcomes based on the evaluation metrics employed, namely accuracy, precision, and recall. The model creation process can be considered complete if these three metrics yield satisfactory results. However, if the outcomes are not deemed satisfactory, we undertake hyperparameter tuning for the CNN model to enhance the training outcomes. For the evaluation of several alternative models, some indicators have been set. These criteria are shown in Table 1.

**Table 1:** Evaluation criteria.

No	Parameter	Indicator
1	Training result	Accuracy, loss, precision, and recall for training and validation, training time
		Evaluation of the possibility of overfitting or underfitting
2	Testing result	Accuracy, precision, recall, and F1 score from overall and each class, testing time
		Evaluation of confusion matrix

Based on Table 1, the best model should have good accuracy, precision, and recall from training and validation datasets with the smallest loss, not show overfitting and underfitting, have a good confusion matrix, and perform the best accuracy, precision, recall, and F1-score on the test results. In addition, the computation time, especially testing (interference time) is also assessed to ensure practicability of the model to be applied in real-world implementations.

### Consent for publication

We hereby provide consent for the publication of the manuscript detailed above, including any accompanying images or data contained within the manuscript.

## Experimental setup

### Dataset

The Emognition dataset contains physiological signals and upper body video recordings from 43 participants who watch movie clips that have been emotionally validated and triggered to produce nine discrete emotions. According to Saganowski et al.^14^ the Emognition dataset offers several advantages compared to other datasets, including nine discrete emotions—amusement, awe, enthusiasm, liking, surprise, anger, disgust, fear, sadness- with one neutral emotion, emphasizing the differences between positive emotions, and enabling diverse analysis in emotion recognition (ER) from the physiology and facial expressions domain. This study only use half-body video data of the Emognition Dataset with a total of 387 videos. There are two types of frame rates: 60 FPS and 30 FPS. For a total, there are 287 video with 60 FPS, while the rest 100 videos are 30 FPS. Generally, the videos in the Emognition dataset vary in length across different videos and classes.

### Data preprocessing

#### Converting video to frame

At this juncture, frames from the video data are extracted by taking into account both the frame rate and the duration of the footage. The Emognition dataset encompasses two distinct frame rates: 60 frames per second (FPS) and 30 FPS. Employing a sampling method, frames at regular one-second intervals are collected throughout the length of the video. Accordingly, for videos recorded at 60 FPS, frames are extracted every 60th frame, and for those at 30 FPS, every 30th frame was selected.

#### Cropping face from frame

Subsequent to frame extraction, we proceed to isolate the facial region in each frame. This segmentation utilizes the Cascade Classifier function from the OpenCV library to precisely delineate the face in every sequence of frames acquired from the previous stage.

#### Data cleaning

According to Saganowski et al.^14^, certain high-intensity emotional expressions manifest under multiple conditions within the film sequences, and a single sequence may elicit multiple emotions. In light of these findings, a subjective data pruning is conducted. Each image is individually inspected, with those deemed suitable retained and the unsuitable ones removed. This pruning process significantly reduces the total dataset volume. Additionally, an absence of a distinct 'surprise' emotion classification lead to its exclusion, narrowing down the emotion categories to nine, including a neutral category. This reduction yields a final count of 2,535 facial images, constituting a mere 6.12% of the original image dataset.

#### Shuffling and splitting

The data randomization and splitting processes are executed simultaneously. Randomized shuffling is managed via the random state parameter, and partitioning is dictated by the specified test size. Random shuffling in train-test splitting is employed to ensure that the training and testing datasets are representative of the overall dataset. This method mitigates the risk of bias in the model training process and enhances the model's ability to generalize from its training to unseen data. By shuffling, the data is randomized, preventing the model from learning potential patterns that may be due to the order of the data rather than the underlying relationships between the variables.

The datasets are then segmented into 80% for training, and 10% each for validation and testing. The aim of this segmentation is to balance the need for a model to learn effectively from the data (requiring a substantial training set) against the need to prevent overfitting and to accurately estimate the model's predictive performance on new, unseen data (requiring separate validation and testing sets). A larger training set allows the model to better understand the complex patterns and relationships within the data, which is vital for developing a model that performs well. Moreover, the relatively high portion of train datasets is carefully determined by considering the limited number of available data at only 2535 facial images.

#### Rescale and resize

Normalization of input pixel values is accomplished through the use of a rescale parameter set at 1/255 within the image data generator, converting the pixel values to a normalized matrix ranging between 0 and 1. Concurrently, resizing of images is administered through the target size parameter, aligning with the predetermined image dimensions for this study, which are set at 300 × 300 pixels.

#### Data augmentation

To further enrich the dataset, we apply data augmentation strategies using an image data generator. This involves applying a series of transformations to the input images, such as height shifts, shear intensity variations, zoom alterations, and horizontal flipping. These modifications enable the generation of new image variants, thereby enhancing the diversity of the training dataset.

### Parameter configuration and model implementation

There are several hyperparameters of the training process that are pre-determined. Details of these parameters are shown in Table 2. Meanwhile, the number of epochs for full learning model is determined based on the Taguchi experiments. During the training, the same hyperparameters setting are set for both transfer learning and full learning models to ensure fair comparison, except the image size which follows the input size of respective architecture and the number of epochs. The number of epochs for transfer learning models are 100 epochs, which is uniformly divided for each 1st and 2nd scenarios.

**Table 2:** Training hyperparameter.

No	Hyperparameter	Value
1	Batch size	32
2	Image size	300 × 300
3	Loss function	Categorical Crossentropy
4	Optimizer	Adam
5	Learning rate	0.0001
6	Evaluation metric	Accuracy, precision, and recall

The proposed method is developed and implemented using TensorFlow written in Python. The models are fully trained and tested using Google Colab, accessed through a computer with Intel(R) Core(TM) i5-10200H CPU with 2.40 GHz, 8 GB RAM, and GeForce GTX 1650 Ti with Max-Q Design.

## Result and discussion

### Model architecture

#### Transfer learning with MobileNet-V2 and Inception-V3

**Figure 5:**
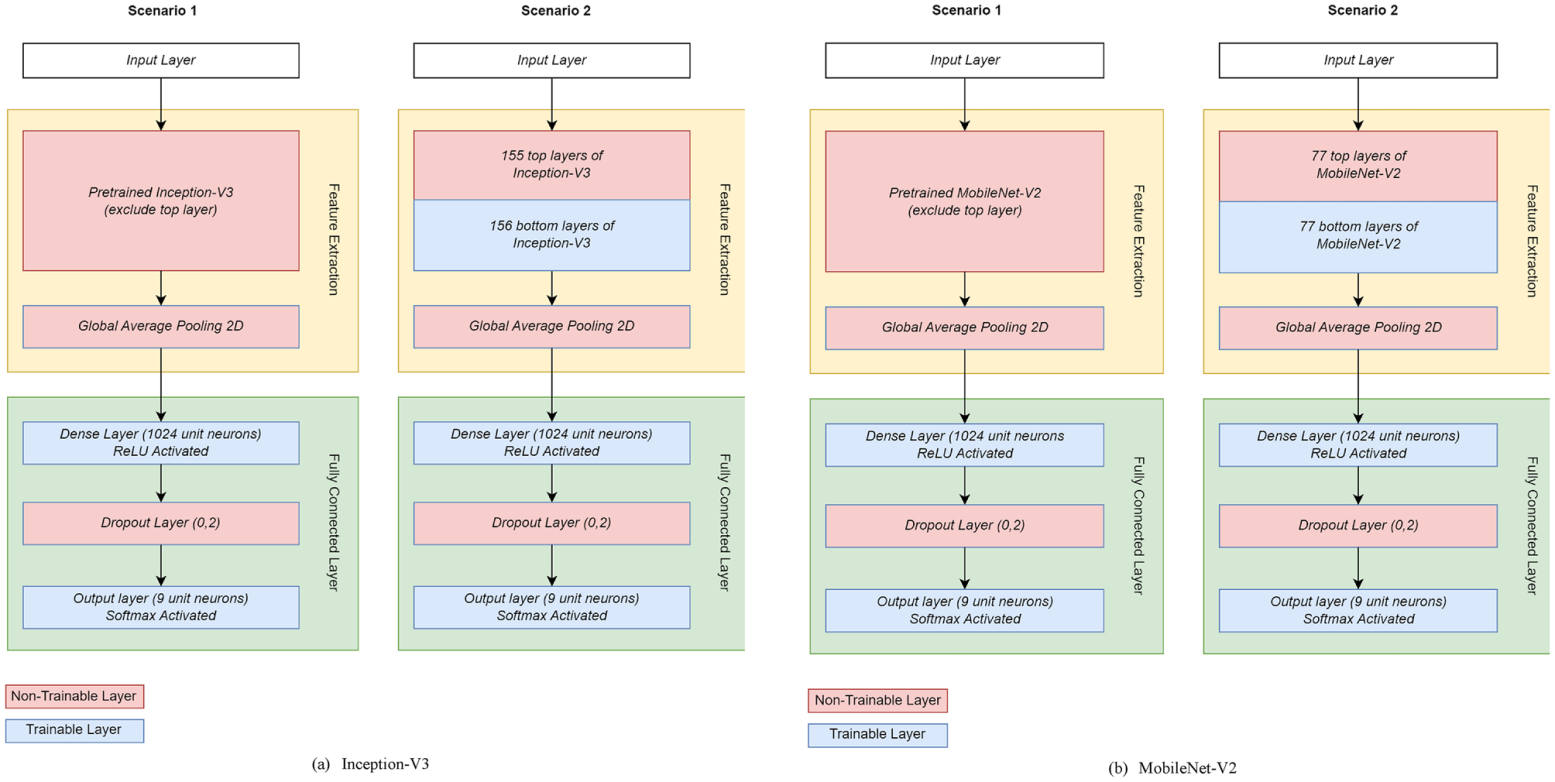
Architectures of transfer learning models.

In the first scenario, we freeze all the layers of pretrained models. This means that the parameters of the pretrained model were not retrained. The input data flowed either forward pass or backward pass through the feature extraction layer without performing any updates. This first scenario allowed the model to train only on the fully connected layer. In the second scenario, we perform fine-tuning by activating the last half of the pretrained layer. The total layers in MobileNet-V2 and Inception V3 are 154 and 311 layers, respectively. Therefore, we activated the last 77 layers in MobileNet-V2 and the last 155 layers in Inception-V3. The Adam optimizer was applied to prevent overfitting by adding a learning rate parameter with 0.0001. The difference in the number of parameters is shown in Table 3, while the illustrations of transfer learning architectures are shown in Fig. 5.

**Table 3:** The differences in the number of parameters.

Transfer learning stage	Parameter type	Architecture	
		Inception-V3	MobileNet-V2
Scenario 1	Trainable	2,107,401	1,320,969
	Non-trainable	21,802,784	2,257,984
Scenario 2	Trainable	18,896,841	3,578,953
	Non-trainable	5,013,344	194,496

#### Full learning model

For the build-from-scratch technique, we used the Taguchi method to find a robust combination between the feature extraction layer and the epoch to be used. The Taguchi method has been widely applied to obtain the optimal architecture design and hyperparameter setting of CNN, thus avoiding time-consuming trial-and-error methods^47^. Here, the options of the number of convolution layers include three levels of 4, 5, and 6 of feature extraction layers. Three of the initial feature extraction layers were identical. The 4th, 5th, and 6th convolution layers were convolution layers with 64 filters, with each filter size being 3 × 3 and using the ReLU activation function. Each convolution layer is followed by a pooling layer with a pool size of 2 × 2. The epoch parameters have levels of 50, 100, and 150 epochs.

**Figure 6:**
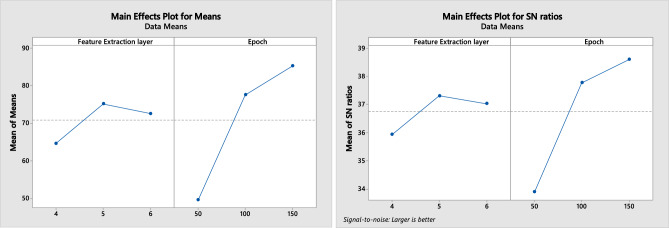
Taguchi results.

The validation accuracy was used for the output, while larger values are better for the type of S/N ratio used. The combination design and its results are obtained as shown in Table 4, while the data analysis output is presented in Fig. 6.

**Table 4:** Taguchi design combination and its result.

Number of feature extraction layers	Number of epochs	Validation accuracy (%)
4	50	43.48
4	100	70.36
4	150	80.24
5	50	52.96
5	100	83.00
5	150	89.72
6	50	52.57
6	100	79.45
6	150	85.77

Based on the main effect plot for the S/N ratio and the main effects plot for the mean in Fig. 6, the robust design combines the number of feature extraction layers at level 5 and the number of epochs at level 150. This combination provides higher accuracy and can produce a system that is not sensitive to changes.

Next, we used five types of convolution layers, each with 16 filters on the first convolution, 32 on the second convolution, and 64 on the third, fourth, and fifth convolutions. The filter size for all convolution layers is 3 × 3. Each convolution layer was followed by a max-pooling layer with a pool size of 2 × 2. We also used the global average pooling layer to convert the feature matrix into a vector for a fully connected layer with two dense layers. The first dense layer had 512 neurons, and the second dense layer (output layer) had 9 neurons, according to the number of classes in the input data model. The activation function used for each layer was ReLU, except for the second dense layer (output layer), which uses Softmax activation because it adapts to the type of problem, which is multiclass classification.

**Figure 7:**
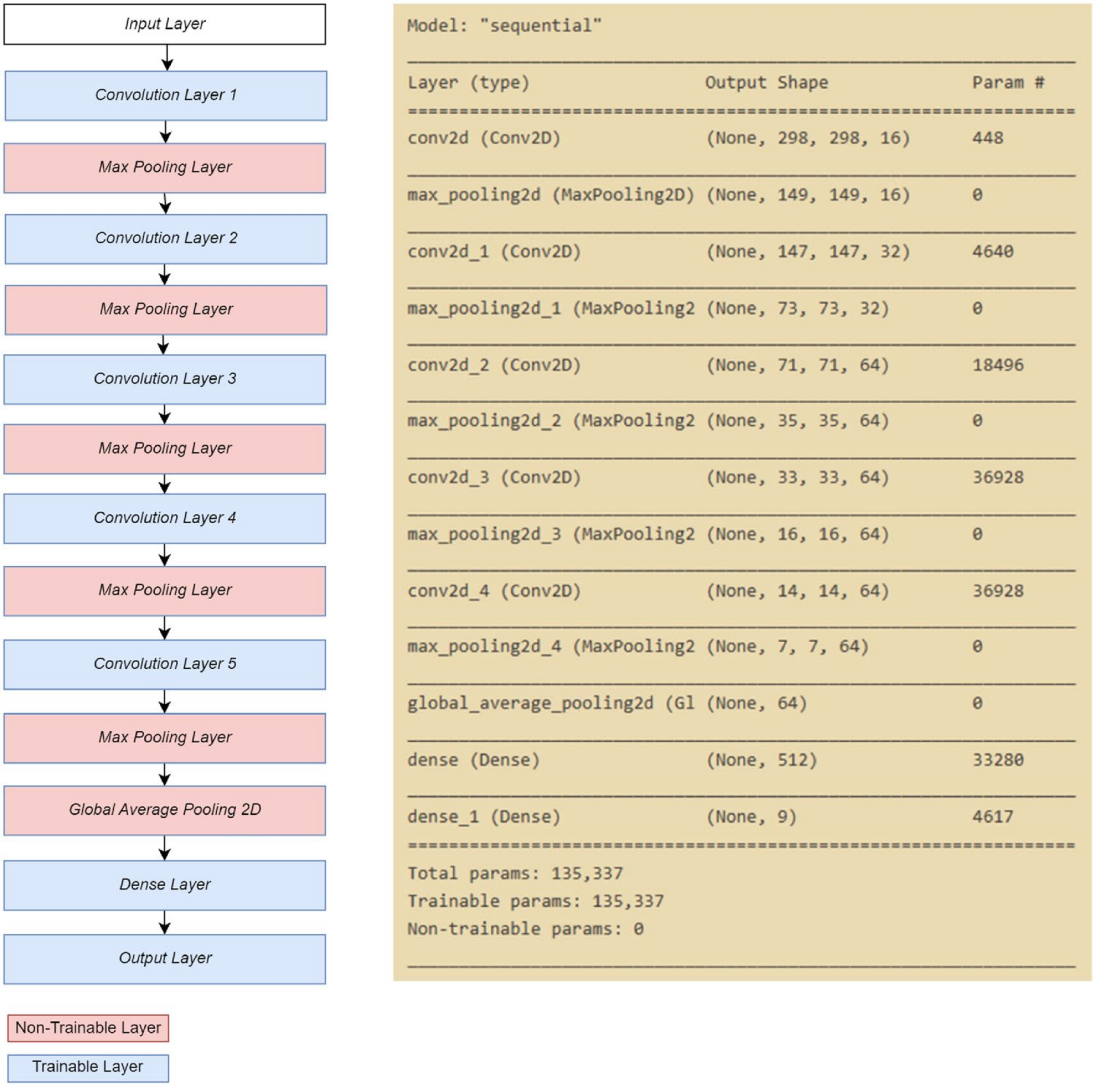
Full learning architecture.

With that architecture, the model had a total of 135,337 trainable parameters. A training process with 150 epochs carried out all these parameters. The architecture of the full learning model is depicted in Fig. 7.

### Training result

**Figure 8:**
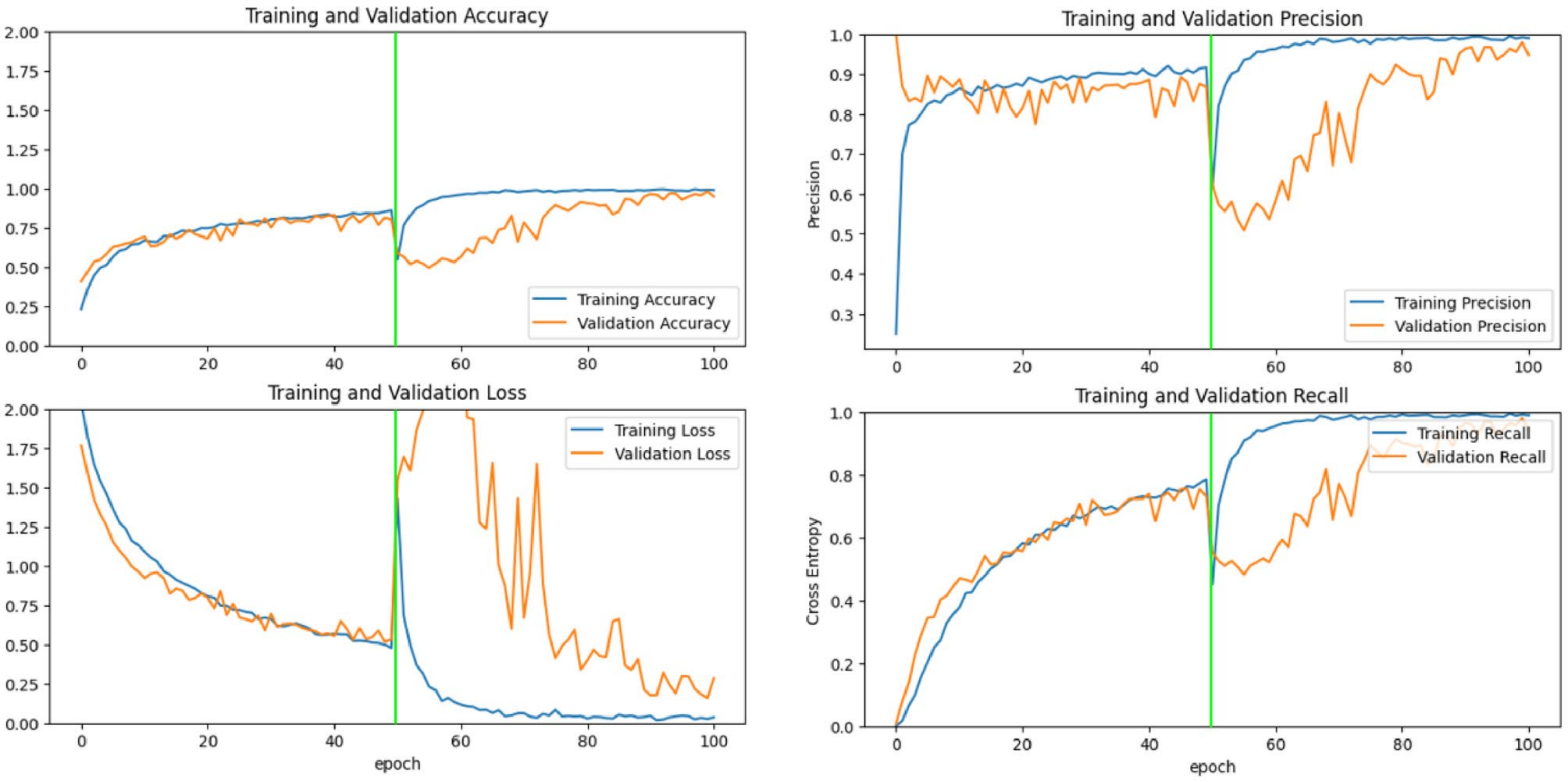
Training process for transfer learning MobileNet-V2 mode.

**Figure 9:**
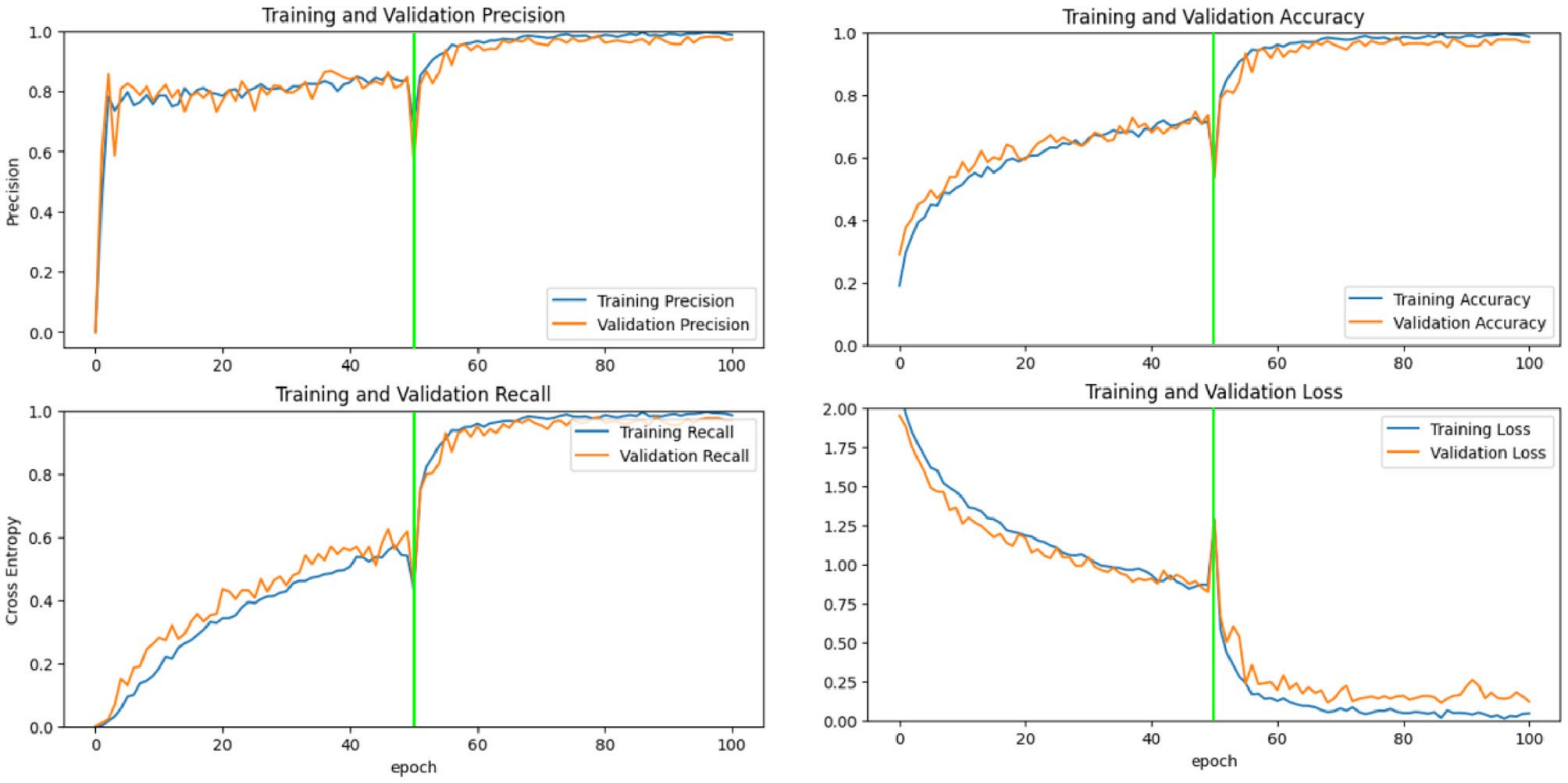
Training process for transfer learning Inception-V3 model.

**Figure 10:**
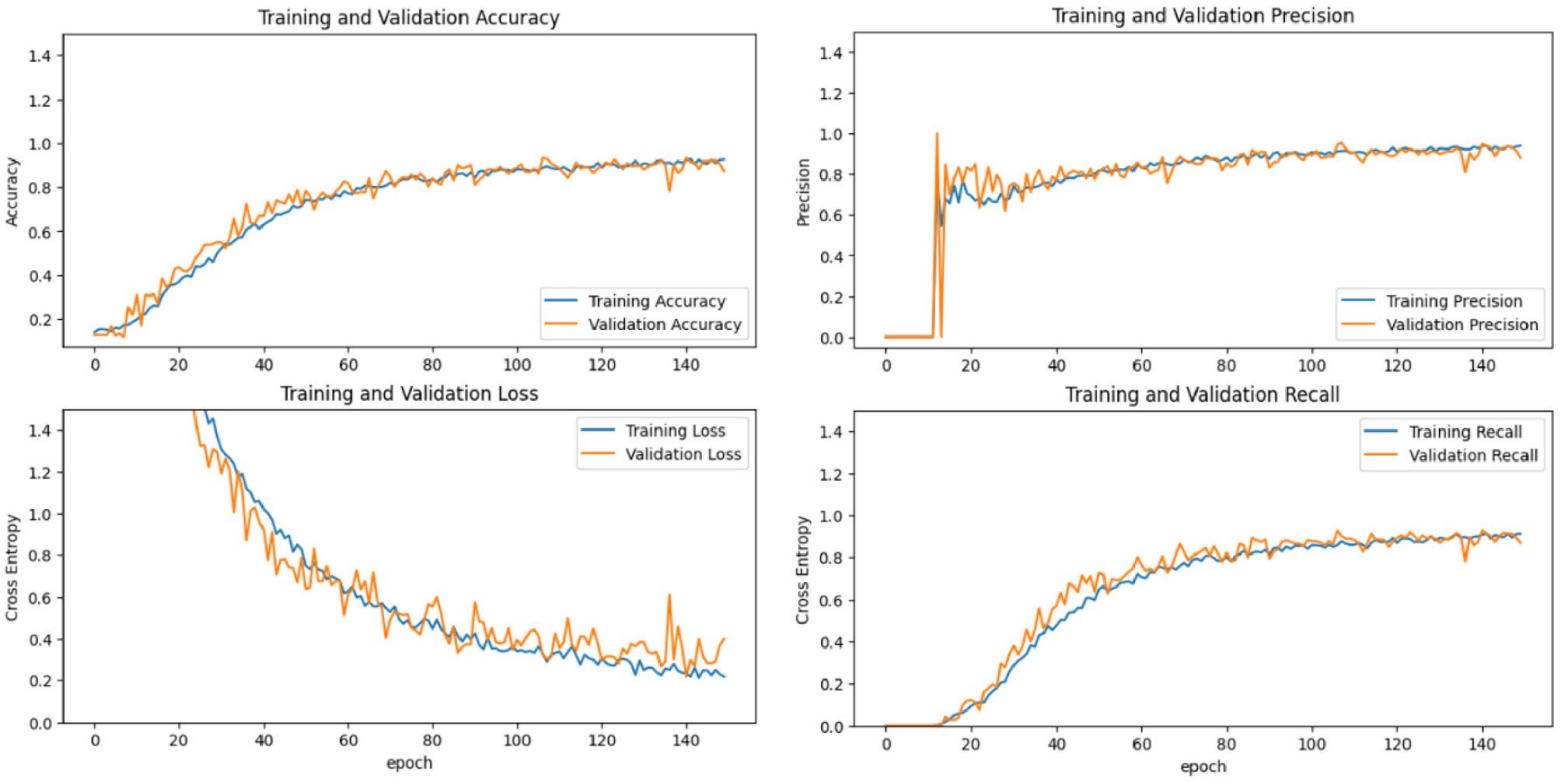
Training process for full learning model.

Training processes were executed using the hyperparameter values described in Sect. “Experimental setup”. Specifically, the number of convolutional layers and epochs for the full learning model was determined based on the Taguchi results. We used two scenarios approach for the transfer learning models during the training process. The training process for the MobileNet-V2, Inception-V3, and full learning models were presented in Figs. 8, 9 and 10, respectively.

The horizontal green line in Figs. 8 and 9 represents the boundary between the first and second scenarios. The training process was conducted for 100 epochs, with each scenario lasting for 50 epochs. Before and after the training process began, the model was evaluated on the validation data. It can be observed that there is a significant improvement in accuracy and a notable reduction in loss. Thus, through the training process using both the first and second scenarios, the model's performance has been successfully enhanced, as evidenced by increased accuracy and decreased loss. This indicates that the model has effectively learned from the training data and can generalize well on the validation data.

Based on the evaluation results on the accuracy, precision, and recall matrices from epoch to epoch on the training and validation data for each type of existing model, it can be concluded that the transfer learning model with Inception-V3 gives the best results until the last epoch. The transfer learning model with Inception-V3 shows a consistent positive trend in the training and validation data without significant fluctuations in the validation data, so there is no overfitting identified. The indications of overfitting and underfitting are also not found on the training graph of MobileNet-V2 and the full learning model, thus indicating proper training for all models.

**Figure 11:**
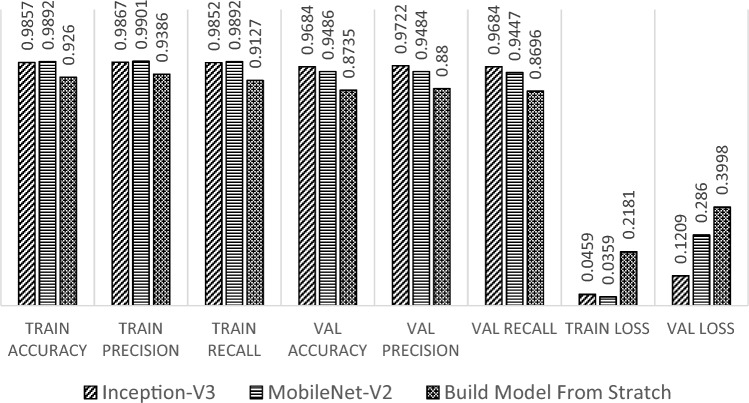
Training results comparison between all models.

**Figure 12:**
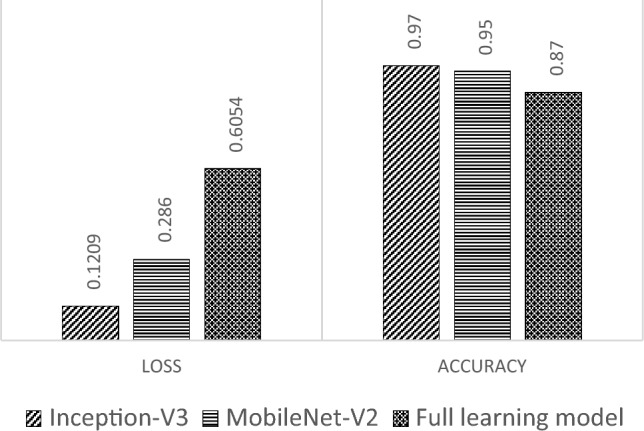
Evaluation of validation data.

Further, the training results on the matrix evaluation of accuracy, precision, recall, and loss for each at the end of the training process can be seen in Fig. 11. Here, the transfer learning model with Inception-V3 can provide optimal results on training data and validation data, the transfer learning model with MobileNet-V2 shows less consistent results between training and validation results. In contrast, the full learning model shows conditions that are less than optimal compared to other models, scoring the lowest precision and accuracy, as well as highest loss both in training and validation dataset. In addition, the evaluation results on the validation data conducted after the training process can be seen in Fig. 12.

**Figure 13:**
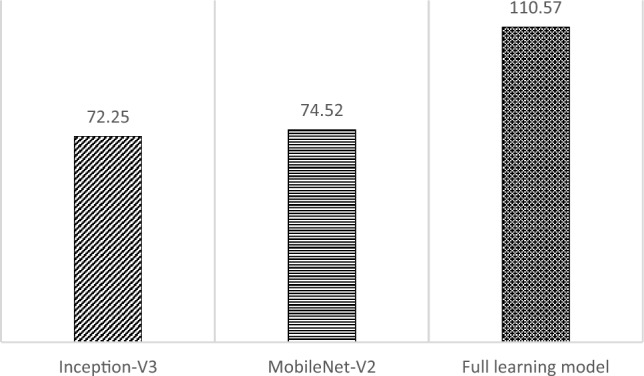
Training time (in minutes) comparison.

Based on Fig. 12, the transfer learning model with Inception-V3 has the highest accuracy value and the smallest loss in the evaluation process using validation data. This model shows the best performance, which indicates that the training process in the transfer learning model with Inception-V3 is more effective than the other models. A comparison of training results is also reviewed from the time of the training process for the three models. The training time comparison is shown in Fig. 13.

Based on Fig. 13, the transfer learning model with Inception-V3 requires a shorter training time when compared to the other models with 72.25 min. Noted that, although Inception-V3 needed longer training time than MobileNet-V2 on the original training using the ImageNet, here the training times for both transfer learning models are not significantly different. Meanwhile, the training time for the full learning model is the longest at 110.57 min. This is because the model requires more time to learn from the beginning with all layers activated, and its model was trained using 150 epochs.

Overall, the transfer learning model with Inception-V3 and the fine-tuning process that has been carried out has produced optimal results in the training process. Fine-tuning in Inception-V3 successfully adapts the model effectively with a shorter training time on the input data.

### Testing results

**Figure 14:**
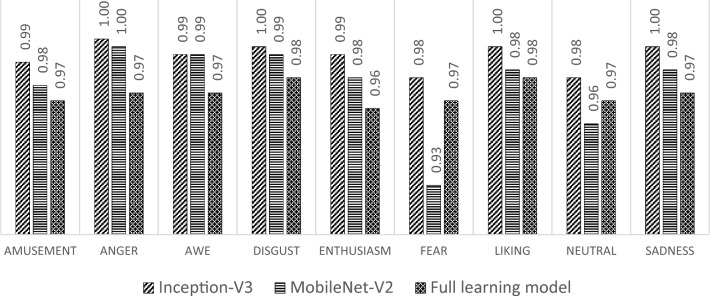
Testing accuracy.

After the training and validation processes are executed, the testing process is conducted to evaluate the performance of the detection model in generalizing new data. Based on the testing process on the same test data, a comparison of the total accuracy results on the test data of the three models that have been built is carried out. The results indicate that the transfer learning model with Inception-V3 has a better total accuracy value than other models, with the accuracy of the transfer learning model with Inception-V3 at 0.96 with MobileNet-V2 at 0.89 and the full learning model at 0.87. This indicates that the ability of the transfer learning model with Inception-V3 to recognize the true class of the entire data is better than the other models. The comparison of the accuracy was also conducted for each class; the results of the comparison are shown in Fig. 14.

Based on Fig. 14, the transfer learning model with Inception-V3 performs better accuracy than other models for each class in the input data. This indicates that the transfer learning model with Inception-V3 can make predictions that generate quite a lot of true positives and true negatives. Only in the awe class does the class accuracy of the transfer learning model with Inception-V3 have the same value as the transfer learning model with MobileNet-V2.

**Figure 15:**
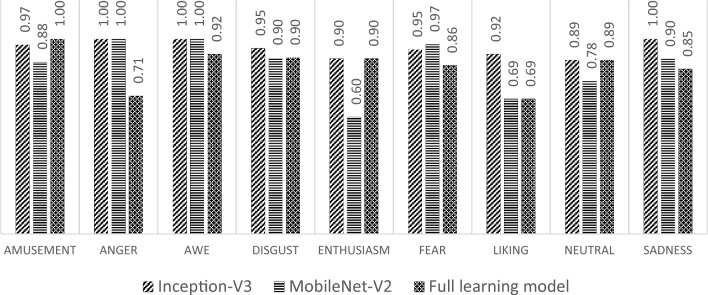
Testing recall.

The comparison of the testing results is also carried out on the recall matrix for the three models that have been built, as shown in Fig. 15. The result indicates that the recall value of the transfer learning model with Inception-V3 is superior in most classes. Only in the fear class the recall of the Inception-V3 model is smaller than the recall from the transfer learning model with MobileNet-V2, whereas in the anger and awe classes, the accuracy of the transfer learning model with MobileNet-V2 and Inception-V3 shows the same value. In the amusement class, the recall of the build model from scratch is superior to the other models, and in the enthusiasm and neutral classes, both recalls are the same. In other classes, the recall of the transfer learning model with Inception-V3 is superior to other models.

**Figure 16:**
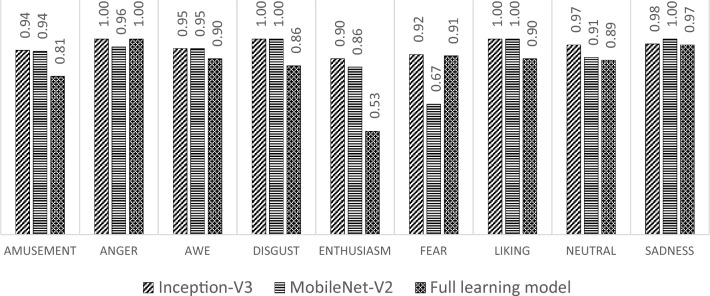
Testing precision.

A comparison of the testing results is also carried out from the perspective of the precision value of each class. The comparison results are shown in Fig. 16. Based on Fig. 16, the precision of the transfer learning model with Inception-V3 dominates in enthusiasm and neutral classes compared to the other models. In the amusement, awe, disgust, and liking types, the precision is the same as the transfer learning model with MobileNet-V3. While in the sadness, the model has lower precision. In the sadness, the precision is dominated by the transfer learning model with MobileNet-V2, and in the anger, precision is the same between the build model from scratch and transfer learning with Inception-V3.

**Figure 17:**
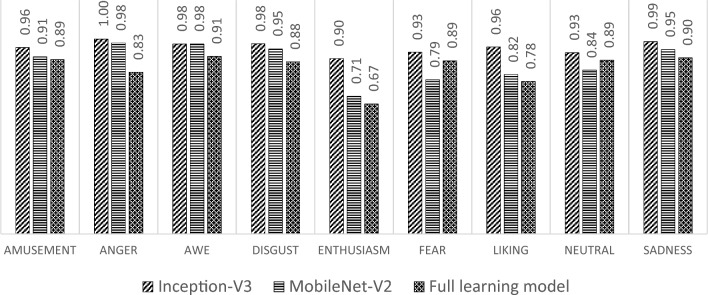
F1-score in testing data.

Through F1-Score, each class's recall and precision values are combined so that a more comprehensive analysis can be obtained. Based on Fig. 17, the transfer learning model with Inception-V3 performs better than other models for each class in the input data. However, in the awe class, the F1-Score value of the Inception-V3 is the same as the F1-Score value of the MobileNet-V2, which means that both have comparable performance in that class.

**Figure 18:**
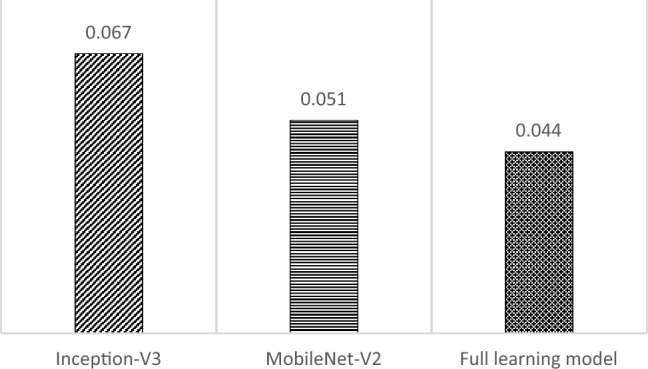
Testing time (in seconds) comparison.

The assessment includes a review of the testing duration for each model when evaluating a single image. The testing time is crucial since it influences the applicability of the developed model to be implemented in the real system. These findings on testing time are depicted in Fig. 18. According to the data presented in Fig. 18, the Inception-V3 model exhibits a longer testing period compared to the other models. This is due to the greater complexity of the transfer learning model with Inception-V3 compared to other models. In contrast to the build-from-scratch model, which has a much simpler model complexity, it has quicker time processing. Nevertheless, the testing time of all models is considerably fast enough to be implemented for real-time detection.

The testing results confirm that the transfer learning model with Inception-V3 is the best choice in dealing with the task of classifying emotions in the Emognition dataset. Better performance on these evaluation matrices indicates the model's ability to capture a balanced overall classification performance and recognize certain emotion classes. Although the transfer learning model with Inception-V3 has a longer testing time, the testing time is not significantly different from other models.

### Experiments on JAFFE and KDEF datasets

To further validate the robustness and versatility of the proposed models, extensive testing was conducted using two well-established facial emotion recognition datasets: the Japanese Female Facial Expression (JAFFE) dataset^48^ and the Karolinska Directed Emotional Faces (KDEF) dataset^49^. These datasets are frequently utilized in the FER field due to their diverse representation of facial emotions and have historically served as benchmarks for evaluating the effectiveness of FER algorithms.

The models used for these experiments were adapted from the best-performing models on the Emognition dataset, as detailed in Sect. “Testing results”. This approach leverages the sophisticated feature extraction capabilities already developed for the Emognition models, thus providing a strong foundation for recognizing emotions in JAFFE and KDEF images. The transfer learning technique was applied, utilizing the same hyperparameter configurations as outlined in Sects. “Model architecture” and “Training result”, ensuring consistency in model training and evaluation.

The performance of the adapted models on the JAFFE and KDEF datasets was assessed based on their testing accuracy and F1-scores. These metrics are critical for comparing the efficacy of the proposed models against existing models documented in recent literature. The results are systematically presented in Table 5, which includes comparative data from other recent studies.

**Table 5:** The test results for JAFFE and KDEF datasets.

Model	JAFFE		KDEF	
	Accuracy (%)	F1-score	Accuracy	F1-score
Proposed models				
MobileNet-V2	91	0.82	92%	0.92
Inception-V3	94	0.92	93%	0.93
Full learning model	91	0.90	89%	0.89
Yang et al.^50^	92.21	–	–	–
Ullah et al.^51^	94	0.81	–	–
Reddi and Khrisna^52^	80	–	–	–
Dada et al.^53^	95.41	0.96	–	–
Sari et al.^54^	86.24	–	82.38%	–
Lasri et al.^55^	97.70	–	86.33%	–
Baygin et al.^56^	98.59	–	94.69%	–

The analysis demonstrates that the proposed models, especially Inception-V3 transfer learning model, are effective for facial emotion recognition tasks across different datasets. When compared to other research, the proposed models are competitive, often outperforming or matching other state-of-the-art results. The model of Dada et al.^53^, Lasri et al.^55^, and Baygin et al.^56^ stand out with slightly higher metrics on JAFFE. However, it should be noted that the proposed models are trained and tuned using the pre-processing and hyperparameters specifically tailored for Emognition dataset, while the existing models were developed and trained for JAFFE and KDEF datasets. The proposed models could be further refined to enhance their accuracy and F1-scores, potentially by incorporating techniques from the best-performing models in the literature or by further tuning their architectures and training parameters according to the respective datasets.

**Figure 19:**
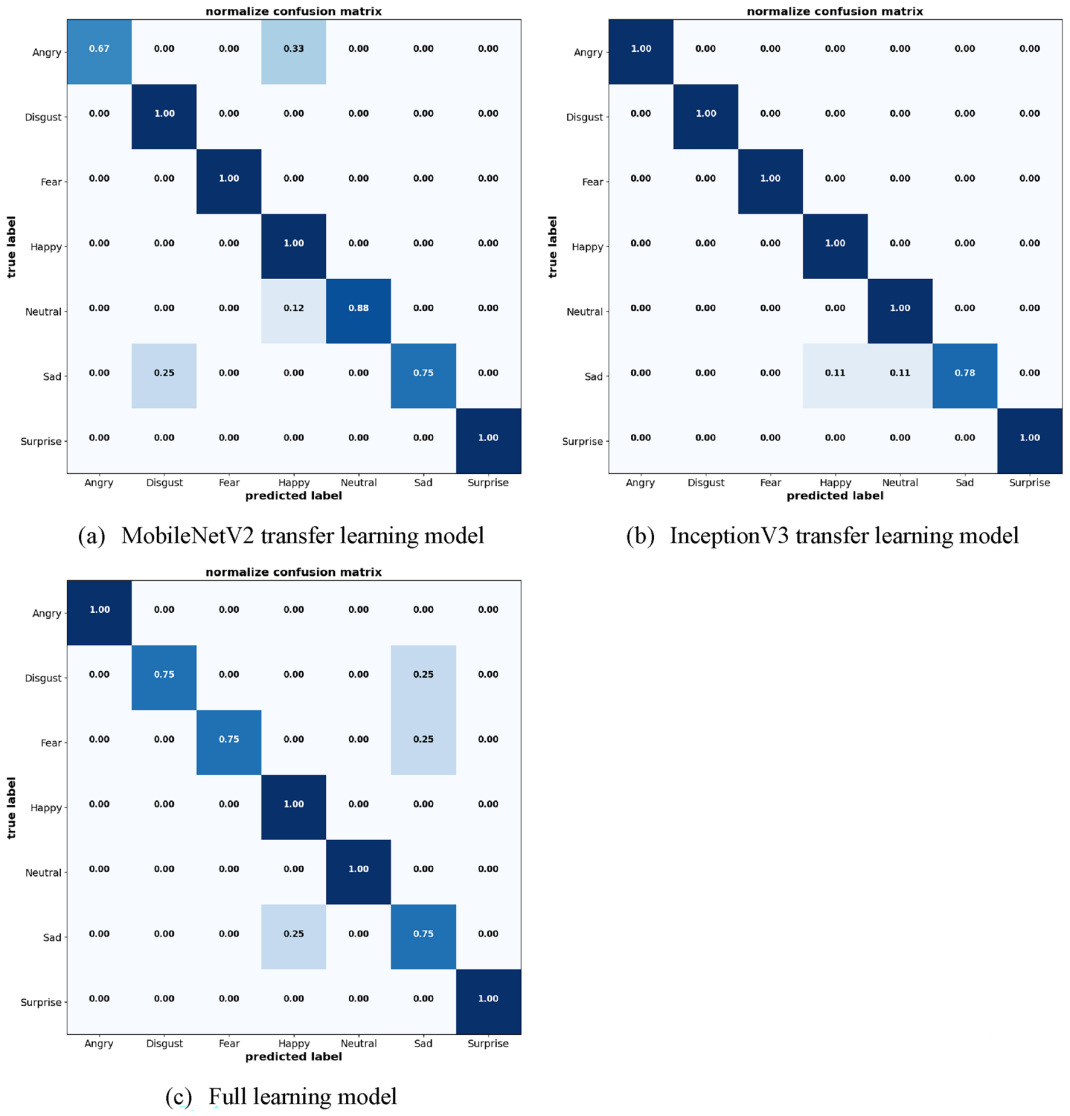
Confusion matrix of the models on JAFFE dataset.

**Figure 20:**
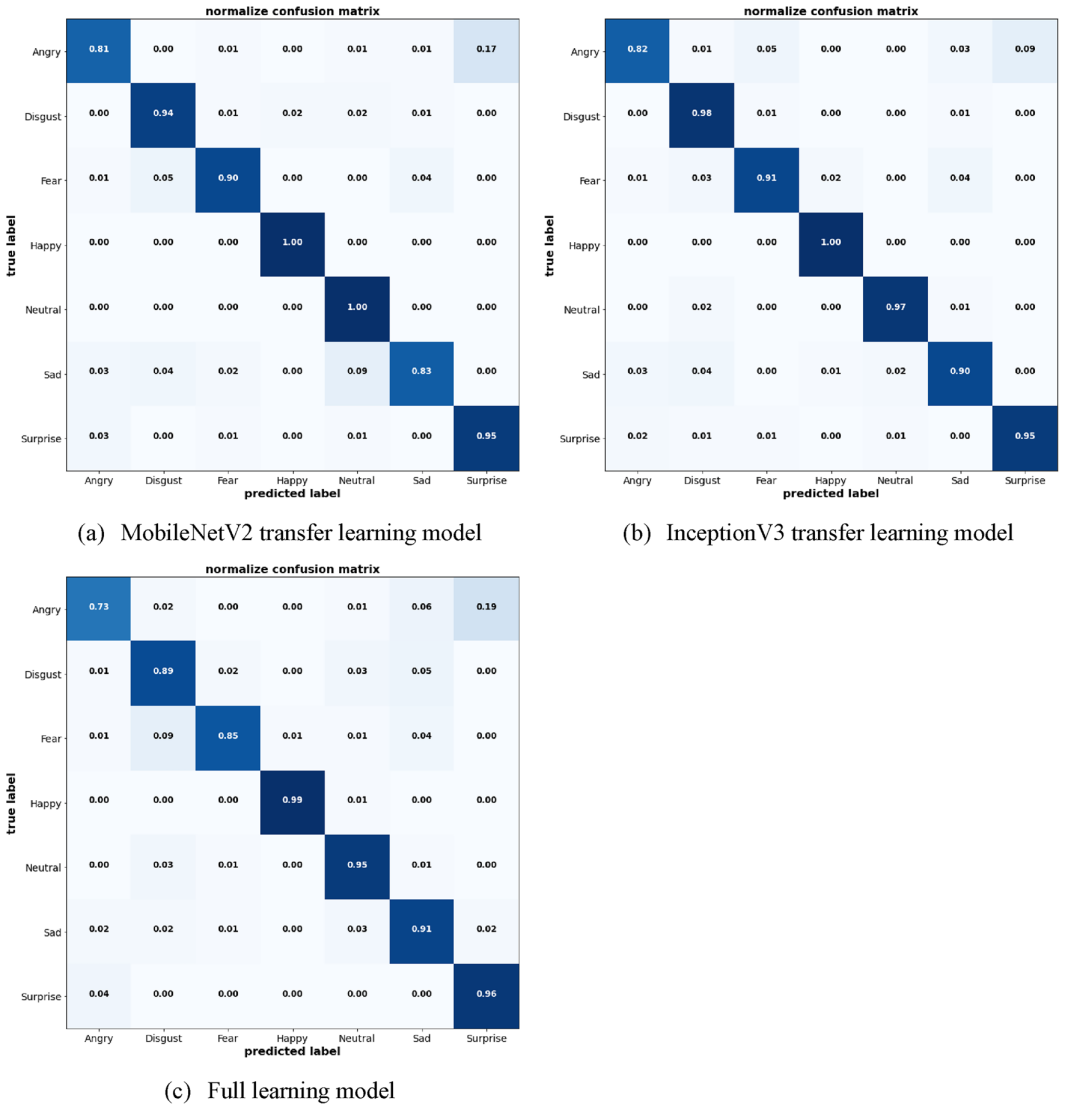
Confusion matrix of the models on KDEF dataset.

To provide a clearer visual representation of model effectiveness, Figs. 19 and 20 display the confusion matrices for the JAFFE and KDEF datasets, respectively. These matrices illustrate the precision of emotion classification across different emotions provided by the datasets, highlighting the models' strengths and areas for improvement in recognizing specific emotional expressions.

The comparative analysis of these models on two different datasets provides important insights into their respective strengths and weaknesses. InceptionV3 emerges as the most consistent and reliable model, particularly advantageous in settings where high accuracy across a diverse range of emotions is required. The MobileNetV2 and Full Learning models, while effective, demonstrate specific areas where enhancement is needed, particularly in the accurate classification of negative emotions. The lower accuracy of MobileNetV2 transfer learning models was notable in sad expression where there is a 25% misclassification rate primarily involving confusion with angry and disgust on the JAFFE dataset. It also showed less precision with angry expression on the KDEF dataset. The full learning model showed some limitations with disgust where it only achieves 75% accuracy with notable misclassifications involving fear and sad on the JAFFE dataset. Meanwhile on the KDEF dataset, the misclassifications primarily involve angry and disgust, indicating a recurring challenge in distinguishing between closely related negative emotions. Further improvement may include fine-tuning and hyperparameter optimization which could offer significant benefits, particularly for models showing potential yet inconsistent performance across emotional categories.

### Discussion

Analyzing the training and testing outcomes shows that the transfer learning model utilizing Inception-V3 outperforms the other models. The transfer learning model with MobileNet-V2 shows less optimal performance compared to the transfer learning model with Inception-V3. This is caused by overfitting after the fine-tuning technique is applied (scenario 2) during the training process. As such, the fine-tuning technique is less suitable when applied to MobileNet-V2 with an Emognition dataset. In addition, the number of parameters used in the training process in the transfer learning model with MobileNet-V2 is less when compared to the transfer learning model with Inception-V3. MobileNet-V2 requires more data to be used in the training process. This is also in line with the finding of Abdulsattar and Hussain^37^, who found that the transfer learning model with MobileNet-V2 had less than optimal results compared to other models.

Although the results indicate that full learning model shows less optimal performance when compared to the overall transfer learning model, the difference on testing accuracy with MobileNet-V2 is not significant. Moreover, the full learning model consumes the lowest testing time among the three models. The full learning model has 135,337 trainable parameters, while the MobileNet-V2 transfer learning model has 1,320,969 trainable parameters in scenario 1 and 3,578,953 in scenario 2. This stark difference in complexity results in notably faster training and testing times for the full learning model. Hence, this results indicates that full learning model is still promising enough to be developed for specific task such as for FER. Several improvements in the datasets, architectural design, and training setting can be made to increase the performance of full learning model.

One of the possible reason for full learning model’s ineffectiveness is due to the lack of training data used by the model to start the fitting process from scratch. The limited data for each category within the Emognition dataset may have hindered the full learning model from reaching a high level of accuracy. Transfer learning models, which benefit from pre-training on extensive and varied datasets, are better equipped for identifying and learning new features and patterns. They come with a foundational understanding of class features, simplifying the classification task. To overcome the data scarcity issue, subsequent research could create an expansive database specifically for FER images to train full learning models more efficiently. This is also in line with what was conveyed by Raja Sekaran et al.^13^, where the use of large amounts of data is needed to support success in creating a CNN model from scratch. Moreover, future research could aggregate existing datasets to form a comprehensive and sizable dataset for this purpose.

Another possible reason could be that the hyperparameters employed were not ideally suited for the problem addressed in this research. Although this study has considered determining the optimal value of number of convolutional layers and number of epoch, there are several other hyperparameters that the optimal values can be explored, such as number of filters, filter sizes, number of neurons, activation functions, regularization, learning rate, and batch size. As such, comprehensive experimentation on these hyperparameters would be necessary.

In addition, improving architectural design of full learning model can be explored by several approach. Normalization techniques like batch or layer normalization can be instrumental in stabilizing the training process, thereby accelerating convergence. To combat overfitting and foster generalization, dropout layers or regularization methods can be strategically integrated into the model. The use of residual connections, inspired by architectures such as ResNet, can be added for allowing the training of deeper networks by enabling the direct flow of gradients. Ensemble methods, which combine the strengths of various models or architectures, can also be employed to improve overall accuracy. Incorporating the aforementioned enhancements into subsequent research has the potential to pave the way for the development of FER models that are not only more sophisticated and precise but also offer greater clarity in their decision-making processes.

Further, this study posseses several limitations that could provide opportunities for future development. The developed models are based on static images for making predictions. The use of static images has several disadvantages as it lacks of temporal context in which static images do not capture the dynamic nature of facial expressions, missing out on temporal cues and changes over time that can be critical for accurately recognizing emotions. In addition, real-world emotional expressions often involve subtle movements and transitions. Static images cannot fully represent these micro-expression, potentially leading to oversimplified models that struggle with the complexity of real facial expressions. A single static image may also not adequately represent the range of expressions associated with a particular emotion, leading to reduced generalizability of the model. Therefore, the future studies can be directed for developing video-based deep learning model for FER. Utilizing video datasets offers a significant benefit by recording facial expressions as they evolve over several frames, delivering a richer, more detailed basis for emotion classification than what static image datasets can provide. This dynamic capture of facial changes enhances the model's ability to accurately recognize a wider range of emotions^57^. Based on the analysis on the JAFFE and KDEF datasets, the proposed models offer promising performance. Hence, future studies can further implement and evaluate the proposed architecures in several other datasets to assess the robustness of the model, especially its ability to learn different emotions.

This study utilizes a form of CNN that is unexplainable, meaning the internal decision-making process of the model is not transparent to the researchers. Future research could focus on explainable artificial intelligence (XAI), focusing on interpretability enhancement to understand how the model processes and classifies input images. The implementation of Explainable Artificial Intelligence (XAI) for Facial Emotion Recognition (FER) holds particular significance for critical and sensitive sectors like police investigations, psychology, judiciary, and healthcare due to several key reasons. In sensitive applications, understanding how decisions are made is crucial for establishing trust. XAI provides transparency into the decision-making process of FER systems, enabling stakeholders to comprehend why a particular emotion was recognized, which is vital for building confidence in the system’s outputs. In environments like judiciary or police investigations, the accuracy of emotion recognition can have profound implications. XAI helps ensure that the conclusions drawn are based on valid, understandable reasoning, which is essential for accountability, especially in legal contexts where decisions can affect the outcomes of cases or investigations. The ethical use of FER in psychology and healthcare requires careful consideration of privacy and consent, as well as the potential consequences of misinterpretation. XAI enables a deeper scrutiny of the ethical implications of deploying such technology by making the operational logic accessible and comprehensible.

## Conclusions

This study introduces an image-based computer vision approach for developing deep learning techniques to automate Facial Emotion Recognition (FER) using the Emognition dataset. The dataset consists of ten expression—amusement, awe, enthusiasm, liking, surprise, anger, disgust, fear, sadness, and neutral. At first, the dataset is subjected to a series of preprocessing steps to transform it into a clean dataset containing 2535 facial images. Subsequently, this dataset is split into 2028 images for training, 253 for validation, and 254 for testing. The development of CNN models involves two distinct methods: transfer learning with fine-tuning using pre-trained models Inception-V3 and MobileNet-V2 and the creation of a CNN model from scratch.

The experimental results demonstrate that all three proposed CNN models perform admirably in classifying emotions across the 9 emotion classes. The training and testing outcomes consistently support the conclusion that the transfer learning model, specifically Inception-V3, exhibits superior performance compared to the other models. This finding also underscores the effectiveness of the fine-tuning process applied to Inception-V3 in adapting the model to the input data. The detail analysis also indicates that the developed models succesfully predicted several new emotions unique in Emognition Datasets, which are amusement, enthusiasm, awe, and liking with high accuracy. Furthermore, this research holds promise for practical implementation in various domains, including marketing, mental health, education, application development, and beyond.

## Data Availability

The datasets in this study are provided by the Emognition team. Please refer to the following article for the datasets: S. Saganowski, J. Komoszyńska, M. Behnke, B. Perz, D. Kunc, B. Klich, L. D. Kaczmarek, and P. Kazienko, “Emognition dataset: emotion recognition with self-reports, facial expressions, and physiology using wearables,” Sci Data, vol. 9, no. 1, pp. 1–9, Dec. 2022, 10.1038/s41597-022-01262-0.
